# Facilitators and barriers to preparing and offering whole grains to children diagnosed with prediabetes: qualitative interviews with low-income caregivers

**DOI:** 10.1186/s12889-021-10915-5

**Published:** 2021-05-17

**Authors:** Tashara M. Leak, Navika Gangrade, June Tester

**Affiliations:** 1 Cornell University, Division of Nutritional Sciences, 416 Savage Hall, Ithaca, NY 14853 USA; 2 UCSF Benioff Children’s Hospital Oakland, 747 52nd Street, Oakland, CA 94609 USA

## Abstract

**Background:**

The prevalence of U.S. youth with prediabetes and type 2 diabetes (T2D) is increasing, with those from racial/ethnic minority and low socioeconomic status (SES) backgrounds at greater risk. Dietary fiber (e.g., whole grains and vegetables) is shown to be inversely associated with T2D risk, yet dietary recommendations are not being met. Caregivers play an important role in home food availability, but low SES neighborhoods are shown to have limited access to fiber-rich foods such as whole grains. The overall aim of this qualitative study was to assess caregiver perceptions about facilitators and barriers to preparing and offering whole grains that they received as part of the 16-week Food Overcoming Our Diabetes Risk (FoodRx) pilot study.

**Methods:**

A convenience sample of 60 youth (8–17 years) with obesity and prediabetes were recruited from an urban pediatric weight management clinic to participate in the FoodRx pilot study. Caregivers accompanied youth to a baseline clinic visit and completed a survey that asked about individual and household characteristics. Exit interviews were conducted at the follow-up clinic visit with caregivers of all youth who completed the study (*n* = 48) in order to assess facilitators and barriers experienced when preparing and offering whole grains. Interview transcripts were coded using the constant comparative method and grounded theory approaches.

**Results:**

Caregivers (*n* = 48) had a mean age of 43 years and were primarily female (*n* = 46) and Hispanic (71%). Main facilitators to preparing and offering whole grains in the home were caregivers’ improved knowledge of whole grain health benefits and the development of strategies to encourage their children to consume whole grains (i.e., pairing whole grains with another liked food). A main barrier for caregivers was the lack of resources available to identify and prepare the novel whole grains that they received.

**Conclusion:**

Findings suggest that caregivers are receptive to incorporating more whole grains into home-prepared meals, but they may need additional nutrition and cooking education to improve their self-efficacy.

**Supplementary Information:**

The online version contains supplementary material available at 10.1186/s12889-021-10915-5.

## Introduction

There is a growing epidemic of prediabetes and diabetes among children and adolescents. Through examining data from the National Health and Nutrition Examination Survey (NHANES), May et al. [1] found that the prevalence of U.S. adolescents (12–19 years) with prediabetes/T2D significantly increased from 9 to 23% between 1999 and 2008. Similarly, findings from the SEARCH for Diabetes in Youth Study, a multi-site observational study of diabetic children and adolescents in the U.S., suggest that the incidence of T2D among youth increased significantly from 9 cases to 12.5 cases (per 100,000 youth) between 2002 and 2012 [2]. The study also reported that non-Hispanic black and Hispanic youth experienced a significant increase in the rate of new cases of T2D between 2002 and 2012, whereas non-Hispanic white youth did not. There is evidence suggesting that the prevalence of T2D may be higher in youth residing in low socioeconomic status (SES) households compared to those residing in higher-income households [3], but additional research is needed to elucidate the relationship between household SES and T2D risk amongst youth.

Dietary fiber (e.g., whole grains and vegetables) plays a significant role in the prevention and treatment of T2D. Especially important is the intake of whole grains, which have been shown to improve glucose control. In a systematic review and meta-analysis of 13 studies on whole grain intake and T2D risk, high intake of whole grains was inversely associated with T2D risk [4]. Results revealed that as whole grain intake increased (up to 50 g/day), T2D risk decreased by 25% [4]. For a 2000 cal diet, the 2015–2020 Dietary Guidelines for Americans recommends consuming 6-oz equivalents (oz. eq.) of grains/day with at least half of those grains being whole grains (i.e., 3-oz. eq./day; 1-oz. eq. = 1 slice of 100% whole wheat bread; USDHHS and USDA, 2015). An oz. eq. is approximately 16 g of whole grains [5]. In general, adolescents in the U.S. are not meeting dietary recommendations for whole grain intake, with recent data suggesting that adolescents consume 0.8-oz. eq. of whole grains on any given day (Tester, Leung, Leak, & Laraia, 2017). Furthermore, socioeconomic disparities in whole grain intake exist, such that adolescents residing in lower income households consume an average of 0.5-oz. eq. per day, while adolescents from a higher socioeconomic background consume an average of 1.0-oz. eq. per day [6].

There are many factors that influence youth intake of whole grains, with sensory-related characteristics (e.g., taste, appearance, and texture) being among the most cited factors and varying depending on the product [7–10]. For example, some studies have shown that youth report favorable liking scores for pizza made with whole grain pizza crust [11, 12], whereas the findings are more mixed regarding the acceptability of whole grain tortillas [13, 14]. To address sensory-related barriers, several studies have examined the effectiveness of partially substituting refined grains with whole grains [11, 12, 14].

Caregivers also play a significant role in whether or not their children consume whole grains based on their own attitudes and beliefs about whole grains, willingness to role model whole grain intake, and whether they make whole grains available and accessible within the home [15, 16]. Several cross-sectional studies have shown that household availability of whole grains is positively associated with whole grain intake among children and adolescents [10, 17, 18]. However, limited neighborhood availability of whole grains continues to be a barrier [9], as it is challenging for caregivers to offer whole grains in the home if whole grains are not available for purchase in the neighborhood. Furthermore, studies have reported that low SES neighborhoods have limited access to healthier foods (e.g., whole grains) [19], and when healthier foods are available, they are more expensive in low SES neighborhoods compared to higher SES neighborhoods [20, 21].

The overall aim of this paper is to assess caregiver perceptions about facilitators and barriers to preparing and offering whole grains that they received as part of the 16-week Food Overcoming Our Diabetes Risk (FoodRx) pilot study. For the FoodRx pilot study, low SES households with a child with obesity and prediabetes received home deliveries of whole grains (bi-weekly) and vegetables (weekly). The FoodRx pilot study addressed the issue of limited neighborhood availability of whole grains, but what remained unclear were facilitators and barriers caregivers experienced when preparing and offering whole grains in the home.

## Methods

### The FoodRx pilot study

A convenience sample of 60 youth (8–17 years) with obesity (body mass index ≥95th age- and sex-percentile) and prediabetes (glycated hemoglobin (HbA1c) 5.7–6.4% or fasting glucose 100–125 mg/dL in the past year [22]) were recruited from an urban pediatric weight management clinic to participate in the FoodRx pilot study. This type of sampling technique is widely used in pilot studies of clinical trials, given its ease and cost-effectiveness [23]. Additional study inclusion criteria required that youth be enrolled in a public insurance program (proxy for low-income status), reside in Oakland or Hayward California, and speak English or Spanish.

Child/caregiver dyads attended a baseline clinic visit where they provided consent or assent, anthropometric and clinical measurements, survey data, and a dietary recall (child only). In addition, research staff discussed the process of home delivery of whole grains and vegetables with caregivers, and caregivers received a binder with educational materials and recipes. After the baseline clinic visit, two additional dietary recalls were collected via telephone from the child. Once the three dietary recalls were collected, households started to receive bi-weekly deliveries of whole grains and weekly deliveries of vegetables for 16 weeks.

A local food bank provided the whole grains and a local farm provided the vegetables. Staff from the local farm picked up the whole grains from the local food bank, combined the items, and delivered them to participants’ homes. In each delivery, at least 1-oz. eq./day of whole grains per person residing in the household was included. During the course of the 16 weeks, households received barley, brown rice, quinoa, whole grain bread, whole grain cereal, whole grain crackers, whole grain pasta, and whole wheat tortillas. Caregiver/child dyads were also invited to attend three optional cooking classes held by the local public health department, and caregivers received weekly phone calls/text messages from research staff to confirm that deliveries were received and to answer any questions about how to prepare foods included in the deliveries. Additionally, instructional videos on how to prepare whole grain recipes were created, and links to these videos were texted to participants.

At the conclusion of the study, caregiver/child dyads attended a follow-up clinic visit that mirrored the baseline clinic visit protocol. At the follow-up clinic visit, caregivers were also invited to participate in a qualitative exit interview. Findings regarding the impact of the FoodRx pilot study on child liking and intake of delivered whole grains and vegetables, as well as on anthropometric and clinical measures (e.g., HbA1c), will be presented in another paper.

### Qualitative exit interviews

Sixty caregiver/child dyads were enrolled in the FoodRx pilot study at baseline, but only 48 caregiver/child dyads participated in the follow-up clinic visit, and thus 48 caregivers participated in the qualitative exit interview. Individual qualitative exit interviews explored the feasibility of preparing and offering at home the whole grains that were included as part of the study. This included the facilitators and barriers that caregivers experienced. More specifically, the semi-structured interview guide was developed to assess the following: 1) factors that influenced caregiver’s intentions to prepare and offer whole grains, 2) household reactions to novel whole grain foods, and 3) whole grain foods that were liked and disliked. The interview guide was reviewed by the FoodRx pilot study co-investigator, who has extensive experience conducting qualitative research with racially/ethnically diverse low-income individuals, and it was pre-tested with other researchers and healthcare providers who work with this patient population. The full interview guide can be found in Additional File 1.

All interviews were conducted in English or Spanish, based on caregiver preference, between May and October 2017. Interviews were conducted by 3 trained research staff, including the principal investigator of the FoodRx pilot study who has experience collecting and publishing qualitative research. Interviews were conducted at the end of each follow-up clinic visit in a private room. Children were present, but all interview questions were directed at the caregivers. Interviews were on average 45 min, audio recorded, translated from Spanish to English, and transcribed verbatim by a professional transcription service called Verbal Ink [24].

Incentives were provided for caregivers’ attendance at clinic visits for the FoodRx pilot study (a $25 gift card at baseline and at follow-up), with no additional incentive for the completion of the exit interview portion of the follow-up visit. The study protocol was approved by the Benioff Children’s Hospital Oakland Institutional Research Board.

### Data analysis

The constant comparative method [25] and grounded theory approaches [26] were used to manually code the qualitative interview transcripts and to identify emergent themes once all 48 interviews were conducted and transcribed. Open coding was implemented whereby two researchers (TML & NG) met in person and independently coded three transcripts. Once they finished coding the three transcripts, they discussed their respective codes and reconciled coding discrepancies. They then independently open coded the remaining 45 transcripts. Once all 48 transcripts were open coded the two researchers met to discuss their respective codes and coding discrepancies were reconciled. The final stage was axial coding, where researchers examined the relationship between codes and grouped codes that were conceptually related into categories. Key emergent themes were then identified.

## Results

Caregivers (*n* = 48) had a mean age of 43 years, were primarily female (*n* = 46), were predominantly Hispanic (71%), and approximately half (51%) were employed. On average, households consisted of 5.7 people (range: 2–15, median 5). Caregiver and household characteristics are described in more detail in Table 1.

**Table 1:** FoodRx Pilot Intervention Caregiver and Household Characteristics (*n* = 48)

Characteristics	Caregivers and Household characteristics *n* = 48 (unless otherwise indicated)
Caregiver age, mean years (range)	43 (28–76)
Caregiver sex, n (%)	
Male	2 (4.17%)
Female	46 (95.83%)
Caregiver race/ethnicity, n (%)	
Non-Hispanic White	1 (2.08%)
Hispanic	34 (70.83%)
Non-Hispanic Black	12 (25%)
Asian	1 (2.08%)
Caregiver Employment Status, n (%)^a^	
Not employed outside home	23 (48.94%)
Employed part-time	15 (31.91%)
Employed full-time	9 (19.15%)
Number of people (adults and children) living in the household, mean n (range)^a^	6 (2–15)
SNAP Participation, n (%)^a^	
Currently	24 (51.06%)
In the past	14 (29.79%)
Never	9 (19.15%)

^a^*n* = 47

**Fig. 1:**
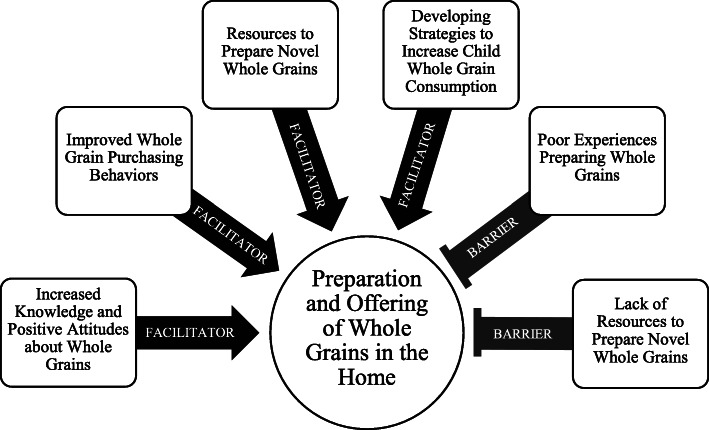
Facilitators and Barriers to Preparing and Offering Whole Grains Among Low-Income Caregivers with a Child with Obesity and Prediabetes: A Conceptual Framework

When caregivers reflected on the feasibility of preparing and offering the whole grains that were included in their FoodRx deliveries, they described several facilitators and barriers. These are discussed in more detail in the following sections. In addition, a conceptual framework summarizing facilitators and barriers is depicted in Fig. 1, and additional supporting participant quotes can be found in Table 2.

**Table 2:** Facilitators and Barriers to Preparing and Offering Whole Grains: Example Quotes from Caregivers with a Child with Obesity and Prediabetes

**Facilitators**	**Example Quotes**
Increased Knowledge and Positive Attitudes about Whole Grains	“I learned a lot about whole wheat foods. To be honest, it was useful even as a family lesson, because we all learned. We learned to read labels.” “From now on, I’m going to keep eating the whole grain instead of eating the food that’s not healthy, and it’s very important for the children nowadays because a lot of them have diabetes, high blood pressure. He′s eight years old, and I do not want him to get that.”
Improved Whole Grain Purchasing Behavior	“When we go shopping now, I read the contents more, especially for bread, things for the sugar content and the grains. Because a lot of things that have the grains actually have a lot of sugar. So that’s something I watch now, the sugar content and the sodium.”
Resources to Prepare Novel Whole Grains	“I was using Pinterest before, and I just found it easier to try and kind of Google things that would make less salt, less seasoning but still more flavor for certain things like the barley.”
Developing Strategies to Encourage Child Whole Grain Consumption	“What I found easier was to implement the whole grains, such as, the quinoa, in recipes that we normally eat. Like if I made a salad, “Oh, we should put quinoa [in it].”” “At first, nobody wanted any [whole grain cereal]. I don’t remember, but it was one they gave me. Everybody said, “No, I don’t want any,” and I told them, “It’s good,” and he tried it and now, he likes it.” “Quinoa is something I didn’t know about, I made it a few times and I tried it and I liked it and I gave it to them. They didn’t like it very much, but they tried it. And it was something new for me because I didn’t know about it, but it’s tasty, it’s good.”
**Barriers**	**Example Quotes**
Poor Experiences Cooking Whole Grains	“Trying to get our wheat noodles to cook without breaking apart to keep its little wheel shape, oh my goodness, I’m like “okay.””
Lack of Resources to Prepare Novel Whole Grains	“I still have a rice that is like dark, like Indian? That one I did not know how to make it, so I have still not made it.”

### Facilitators

#### Increased knowledge and positive attitudes about whole grains

A facilitator to preparing and offering whole grains in the home was caregivers’ increased knowledge and positive attitudes about them. Overall, caregivers stated that they learned more about whole grains as a result of participating in the FoodRx pilot study. There were discussions about being about to distinguish between whole grains, multigrains, and refined grains after participating in the FoodRx pilot study. For example, one caregiver stated, “Now I know that multigrain is not whole wheat.” Additionally, almost all participants referred to whole grains as “healthy,” but fewer participants spoke about specific nutrition and health benefits of whole grains. One caregiver acknowledged that whole grains increase satiety: “… with a little bit you get full … so you don’t even have to eat a lot of it … it’s like you end up satisfied faster.” There were also comments about whole grain intake being associated with improved weight status. For instance, one mother stated, “What I also like is that it helps us to maintain a healthy weight.” Lastly, there were conversations about how caregivers perceived their child’s diabetes risk to be improved as a result of them eating more whole grains. One participant explained, “It helped him a lot … the whole wheats and the whole grains, the pasta and the low sugar and the no sugar – it changed him.”

#### Improved whole grain purchasing behaviors

In addition to knowledge and attitudes, caregivers improved their whole grain purchasing habits. Participants discussed being better able to identify products made with whole grains when shopping for food, as well as making changes in their purchasing behaviors. One mother stated that she enjoyed “learning to know what to look for and what to buy and reading labels, like how much fiber does this have.” Most of the caregivers were able to identify at least one whole grain product included in the FoodRx deliveries that their family liked, and they discussed how they planned to continue to purchase that item. The whole grain pasta was one of the most liked items included in the FoodRx deliveries. One participant explained, “… the whole wheat pasta, that’s all we eat now. That’s been one of the biggest major changes that we made.” Families liked other whole grains, as well. For example, one mother stated, “I really liked receiving [whole grain] cereal, which we took pictures of and we plan on continuing to buy them.” Another parent made a broader comment about the FoodRx deliveries, “I’m saying to myself, ‘well why didn’t I buy this before?’ I mean, you know, so it made you start thinking, like, all of this stuff is here but I never once thought about buying it when I seen it in the store … But now I’m buying it, you know, because I really actually like it and my kids like it, too.”

#### Resources to prepare novel whole grains

Caregivers also talked about the resources they used to help them with preparing some of the whole grains that were more novel to them. Several participants stated that they tried recipes from the FoodRx recipe book that they received at the baseline clinic visit, with one mother acknowledging, “Well, they gave me the book [FoodRx recipe book] and it came with recipes for how to prepare the food.” Caregivers also mentioned searching for recipes on the internet and/or asking family or friends for advice on how to prepare certain whole grains that they were less familiar with. Thus, some were able to figure out how to prepare certain whole grains in a way that they and their family would be receptive to. For example, one participant explained that she learned to “cook it [brown rice] the right way because it requires more water than the white rice.”

#### Developing strategies to encourage child whole grain consumption

Another facilitator to preparing and offering whole grains in the home was the development of strategies to encourage their children to eat the whole grains included in the FoodRx deliveries. The most commonly reported strategy was to pair a whole grain with something else that the child already liked. Sometimes whole grains were paired with condiments and/or dips. For example, one participant stated that she saw her child pair foods: “The berry-flavored cereals that were coming with the granolas and things like that, I saw them on top of yogurt.” Sometimes the refined grain version of a food was mixed with the whole grain version of a food. For instance, one mother explained, “In order to eat it [brown rice], we would mix it with white rice, because they can’t stop eating white rice, but we would add brown rice.” Other times, whole grains were mixed with non-grain-based foods as one parent detailed that the whole grains “were easy to just kind of stir fry together and carrots when you throw them in soup or cook with some turkey in the oven.”

Verbal and non-verbal cues were also used to encourage children to eat whole grains. When trying to get her daughter to try Triscuits, one participant told her, “If you don’t try it, you don’t know if you’re going to like them.” Another reported that she would affirm her child by saying things like, “They’re good, right baby?” A few caregivers stated that they and/or other adults in the home would role model eating whole grains. One mother explained, “We do try to eat them [whole grain crackers] for the children to want them, for them to get used to the taste.” Ultimately by discussing their various strategies, caregivers revealed their desire for their children to consume the delivered whole grains.

### Barriers

#### Poor experiences preparing whole grains

A significant barrier to preparing and offering whole grains in the home was poor experiences cooking the whole grains. Caregivers found it especially challenging to prepare whole grains that were novel to them. Several participants explained that when they prepared novel whole grains for the first time, the grains “came out very hard.” Some stated that they never figured out how to prepare some of the whole grains, and so they just stopped trying. For example, one mother said, “It’s really hard for us to do brown rice, maybe more so me because it’s coarse... I cook it and then they waste it, so I just stopped cooking it.” Overall, caregivers expressed frustration with the process of cooking some of the whole grains they received.

#### Lack of resources to prepare novel whole grains

Another barrier to preparing and offering whole grains in the home was the lack of resources to know how to cook them. Several caregivers did not attempt to prepare some of the whole grains included in the FoodRx deliveries, and the most commonly cited reason was because they did not know how to prepare them. One caregiver explained, “Some of the food they sent, the grains, I had never seen before and I didn’t know how to cook some of them.” The one whole grain that generated the most adverse reaction was quinoa with caregivers describing it as “too weird,” saying “I look at it and I just go, ‘ehh’” and remarking that quinoa looks like “frog eggs.”

Additionally, although several participants felt they had access to some resources to be able to help them prepare the novel whole grain, the resources were insufficient and the whole grains were still too novel for them to try to prepare. When one mother was asked why she had not prepared the quinoa she responded, “Because I had never cooked it, I had never cooked it, that’s why. But it came with instructions on how to do it. And also, a lady from work told me, ‘Oh you can just boil it with chicken stock, and it gets flavor.’” Ultimately, a large number of participants expressed difficulty in preparing them due to a lack of adequate resources and support.

## Discussion

Several interventions have attempted to improve fruit and vegetable consumption, but fewer interventions have aimed to improve whole grain intake, especially among vulnerable populations, such as children from low SES backgrounds diagnosed with prediabetes. The overall aim of the FoodRx pilot study was to increase fiber-rich food intake among obese children diagnosed with prediabetes that reside in low SES households by providing deliveries of whole grains and vegetables. Given that caregivers are often responsible for what foods are available and prepared in the home, it was important to conduct this current study in order to understand their perceptions about preparing and offering whole grains to their children.

Previous studies reveal that there is a general lack of knowledge about whole grains and the health benefits of them [7, 27]. For example, focus groups conducted with adults (almost half of whom were parents) demonstrated that participants knew what constitutes a whole grain, but only had a basic understanding of why consuming whole grains are an integral part of a healthy diet [27]. In another study, parents of elementary school-aged children reported that they recognized products if the phrase “whole grains” was written on the packaging and that whole grains were healthier than refined grains, but they were unable to articulate how [7].

Nutrition knowledge is positively associated with dietary intake [28], and thus intervention studies aimed at increasing whole grain intake have attempted to increase whole grain knowledge [16, 29]. For example, Burgess-Champoux et al. [16] engaged caregivers and children in an intervention to increase whole grain intake that included providing whole grains at school and nutrition education to both caregivers and children. At the end of the intervention, whole grain knowledge and intake significantly increased. Similarly, findings from the present FoodRx pilot study reveal that at study conclusion caregivers reported that they had a better understanding that whole grains are healthy, and some were even able to describe specific health benefits of whole grains. These findings may be a result of the study binder provided at the baseline clinic visit that included educational materials about whole grains. As such, this study and previous research highlight the importance of including whole grain education in interventions aimed at increasing whole grain intake.

After the completion of the FoodRx pilot study, many caregivers expressed that they lacked the skills and/or confidence to prepare whole grains that they were unfamiliar with. This is similar to perceptions from adult participants in an intervention by Kuznesof et al. [30], which aimed to increased whole grain intake by having participants substitute non-whole grain foods with whole grain equivalents that were provided. Despite being provided whole grains and instruction on how to substitute them in their diets, participants reported that they lacked the skills necessary to prepare certain whole grain foods and suggested that they had low self-efficacy when it came to preparing whole grains [30]. Other studies have examined what it is like for adults to prepare less familiar vegetables, and these studies report similar findings to whole grains, such that preparing novel vegetables is challenging for adults and it takes time to acquire skills and improve self-efficacy to prepare them [31, 32]. Thus, regardless if caregivers are preparing a novel vegetable or a novel whole grain, research suggests that lack of skills and confidence to prepare novel foods must be addressed. Other studies that have improved skills and self-efficacy for preparing and offering healthy foods have had participants engage in in-person culinary education, including demonstrations and hands-on food preparation [33, 34]. Therefore, it may be useful to incorporate several sessions of formal, in-depth culinary education into future interventions aimed at increasing whole grain intake.

Caregivers in the FoodRx pilot study described strategies they used to encourage whole grain consumption for children in the home, such as pairing whole grains with liked foods and providing verbal encouragement. Many of the strategies that FoodRx caregivers developed are similar to strategies that researchers have tested when attempting to increase child vegetable intake [35–38]. In one study on increasing child whole grain intake, Burgess-Champoux et al. [7] asked parents for advice on how to introduce whole grain foods to children. Some of the suggestions included introducing whole grains gradually, allowing children to sample the whole grain foods, and possibly branding whole grain products with cartoon characters. As such, this current study provides additional information on strategies to increase whole grain intake among children. For example, caregivers in this current study paired whole grains with a liked food and mixed whole grains with refined grain foods. Therefore, it may be useful to describe these strategies in future whole grain intervention materials for caregivers in order to facilitate their offering of whole grains in the home.

This study is not without limitations. Using a convenience sample for the FoodRx pilot study limits the generalizability of these qualitative findings, since the sample may not be representative of all youth with obesity and prediabetes from low SES households. In addition, parental feeding behaviors are naturally different with younger children, and caregivers with children younger than the age included in this study (8–17 years) may have facilitators and barriers for whole grain intake not explored here. Furthermore, because these children had been identified to have prediabetes, caregivers may have had a higher level of concern and motivation than caregivers of peers with comparable sociodemographic backgrounds but lacking the identified risk for diabetes. Caregivers may have also been influenced to speak favorably about whole grains as a result of being in the FoodRx pilot study where whole grains were provided weekly and focused on. In addition, the authors did not concurrently conduct data collection and analysis due to study time constraints, which may have been beneficial in refining the interview guide. Furthermore, additional checks of data trustworthiness (checking transcriptions against recordings, 3rd coder, etc.) were not employed as the interviews were transcribed by a professional service and there were no coding discrepancies that were not easily reconciled. Lastly, due to the small sample size and qualitative exploration, it may be necessary to quantitatively (e.g., surveys) examine the feasibility of preparing and offering whole grains in the home among a larger sample.

## Conclusion

Given the growing number of youth at risk for T2D, it is imperative to identify effective preventative strategies. Research shows that consuming adequate amounts of whole grains can be protective against T2D [4]. However, youth are not meeting dietary recommendations [6]. Caregivers are the gateway to the home food environment and thus play an essential role in whether or not whole grains are available and accessible for youth [15, 16]. However, to our knowledge, no study has examined caregiver perceptions of the feasibility of offering and preparing whole grains in low SES households with a child at risk for a chronic disease. As such, findings from the current study address a major gap in the literature. The qualitative interviews revealed that caregivers are receptive to offering whole grains to their children, but they may need additional support to address barriers to preparing novel whole grains. Future researchers should address these barriers when designing whole grain interventions for households with a child at risk for T2D.

## Supplementary Information

**Additional file 1.** Food Rx Qualitative Interview Guide. English language version of the interview guide

## Data Availability

All data generated and analyzed during this study are available from the corresponding author upon reasonable request.

## Abbreviations

FoodRx: Food overcoming our diabetes risk
T2D: Type 2 diabetes
oz. eq.: Ounce equivalent(s)

## References

[1] May AL, Kuklina EV, Yoon PW (2012). Prevalence of cardiovascular disease risk factors among US adolescents, 1999-2008. Pediatrics.

[2] Mayer-Davis EJ, Lawrence JM, Dabelea D, Divers J, Isom S, Dolan L (2017). Incidence trends of type 1 and type 2 diabetes among youths, 2002-2012. N Engl J Med.

[3] Dabelea D, Stafford JM, Mayer-Davis EJ, D’Agostino R, Dolan L, Imperatore G, Pihoker C (2017). Association of Type 1 diabetes vs type 2 diabetes diagnosed during childhood and adolescence with complications during teenage years and young adulthood. JAMA.

[4] Schwingshackl L, Hoffmann G, Lampousi A-M, Knüppel S, Iqbal K, Schwedhelm C, Boeing H (2017). Food groups and risk of type 2 diabetes mellitus: a systematic review and meta-analysis of prospective studies. Eur J Epidemiol.

[5] Dabelea D, Divers J, Isom S, Dolan L, Imperatore G, Linder B, Marcovina S, Pettitt DJ, Pihoker C, Saydah S, Wagenknecht L, U.S. Department of Health and Human Services and U.S. Department of Agriculture, SEARCH for diabetes in youth study (2015). 2015–2020 Dietary Guidelines for Americans.

[6] Tester JM, Leung CW, Leak TM, Laraia BA (2017). Recent uptrend in whole-grain intake is absent for low-income adolescents, National Health and nutrition examination survey, 2005–2012. Prev Chronic Dis.

[7] Burgess-Champoux TL, Marquart L, Vickers Z, Reicks M (2006). Perceptions of children, parents, and teachers regarding whole-grain foods, and implications for a school-based intervention. J Nutr Educ Behav.

[8] Jervis MG, Jervis SM, Guthrie B, Drake MA (2014). Determining Children’s perceptions, opinions and attitudes for sliced Sandwich breads. J Sens Stud.

[9] Kamar M, Evans C, Hugh-Jones S (2016). Factors influencing adolescent whole grain intake: a theory-based qualitative study. Appetite.

[10] Larson NI, Neumark-Sztainer D, Story M, Burgess-Champoux T (2010). Whole-grain intake correlates among adolescents and young adults: findings from project EAT. YJADA.

[11] Chan HW, Burgess Champoux T, Reicks M, Vickers Z, Marquart L. White whole wheat flour can be partially substituted for refined wheat flour in pizza crust in school meals without affecting consumption. J Child Nutr Manage. 2008;32(1) Available at: https://schoolnutrition.org/5%2D%2Dnews-and-publications/4%2D%2Dthe-journal-of-child-nutrition-and-management/spring-2008/volume-32,-issue-1,-spring-2008%2D%2D-chan;-burgess-champoux;-reicks;-vickers;-marquart/.

[12] Tritt A, Reicks M, Marquart L (2014). Reformulation of pizza crust in restaurants may increase whole-grain intake among children. Public Health Nutr.

[13] Chu YL, Warren CA, Sceets CE, Murano P, Marquart L, Reicks M (2011). Acceptance of two US Department of Agriculture commodity whole-grain products: a school-based study in Texas and Minnesota. J Am Diet Assoc.

[14] Toma A, Omary MB, Marquart LF, Arndt EA, Rosentrater KA, Burns-Whitmore B, Sung A (2009). Children’s acceptance, nutritional, and instrumental evaluations of whole grain and soluble Fiber enriched foods. J Food Sci.

[15] Burgess-Champoux TL, Chan HW, Rosen R, Marquart L, Reicks M (2008). Healthy whole-grain choices for children and parents: a multi-component school-based pilot intervention. Public Health Nutr.

[16] Burgess-Champoux TL, Rosen R, Marquart L, Reicks M (2008). The development of psychosocial measures for whole-grain intake among children and their parents. J Am Diet Assoc.

[17] Lipsky LM, Nansel TR, Haynie DL, Mehta SN, Laffel LMB (2012). Associations of food preferences and household food availability with dietary intake and quality in youth with type 1 diabetes. Appetite.

[18] Rosen RA, Burgess-Champoux TL, Marquart L, Reicks MM (2012). Associations between whole-grain intake, psychosocial variables, and home availability among elementary school children. J Nutr Educ Behav.

[19] Cavanaugh E, Mallya G, Brensinger C, Tierney A, Glanz K (2013). Nutrition environments in corner stores in Philadelphia. Prev Med.

[20] Gustafson A, Hankins S, Jilcott S (2012). Measures of the consumer food store environment: a systematic review of the evidence 2000-2011. J Community Health.

[21] Jetter KM, Cassady DL (2006). The availability and cost of healthier food alternatives. Am J Prev Med.

[22] American Diabetes Association (2018). Classification and diagnosis of diabetes: standards of medical care in diabetes. Diabetes Care.

[23] Elfil M, Negida A. Sampling methods in clinical research; an educational review. Emergency. 2017;5(1). PMC5325924 28286859

[24] Verbal Ink. 11835 W. Olympic Blvd. Ste. 1020E. Los Angeles, CA 90064. www.verbalink.com. n.d.

[25] Strauss AL, Corbin J (2008). Basics of qualitative research: techniques and procedures for developing grounded theory.

[26] Denzin NK, Lincoln YS (2005). Handbook of qualitative research.

[27] McMackin E, Dean M, Woodside JV, McKinley MC (2013). Whole grains and health: attitudes to whole grains against a prevailing background of increased marketing and promotion. Public Health Nutr.

[28] Spronk I, Kullen C, Burdon C, O’Connor H (2014). Relationship between nutrition knowledge and dietary intake. Br J Nutr.

[29] Croy M, Marquart L (2005). Factors influencing whole-grain intake by health club members. Top Clin Nutr.

[30] Kuznesof S, Brownlee IA, Moore C, Richardson DP, Jebb SA, Seal CJ (2012). WHOLEheart study participant acceptance of wholegrain foods. Appetite..

[31] John JH, Ziebland S (2004). Reported barriers to eating more fruit and vegetables before and after participation in a randomized controlled trial: a qualitative study. Health Educ Res.

[32] Shankar S, Klassen A (2001). Influences on fruit and vegetable procurement and consumption among urban African-American public housing residents, and potential strategies for intervention. Fam Econ Nutr Rev.

[33] Hutchinson J, Watt JF, Strachan EK, Cade JE (2016). Evaluation of the effectiveness of the Ministry of Food cooking programme on self-reported food consumption and confidence with cooking. Public Health Nutr.

[34] Overcash F, Ritter A, Mann T, Mykerezi E, Redden J, Rendahl A (2018). Positive impacts of a vegetable cooking skills program among low-income parents and children. J Nutr Educ Behav.

[35] Correia DC, O'Connell M, Irwin ML, Henderson KE (2014). Pairing vegetables with a liked food and visually appealing presentation: promising strategies for increasing vegetable consumption among preschoolers. Child Obes.

[36] Draxten M, Fulkerson JA, Friend S, Flattum CF, Schow R (2014). Parental role modeling of fruits and vegetables at meals and snacks is associated with children's adequate consumption. Appetite..

[37] Faught E, Vander Ploeg K, Chu YL, Storey K, Veugelers PJ (2016). The influence of parental encouragement and caring about healthy eating on children’s diet quality and body weights. Public Health Nutr.

[38] Leak TM, Swenson A, Rendahl A, Vickers Z, Mykerezi E, Redden JP (2017). Examining the feasibility of implementing behavioural economics strategies that encourage home dinner vegetable intake among low-income children. Public Health Nutr.

