# Supplementary material for: Facilitators and barriers to preparing and offering whole grains to children diagnosed with prediabetes: qualitative interviews with low-income caregivers
Source: BMC Public Health. 2021 May 17;21:931. doi: 10.1186/s12889-021-10915-5 (PMC8127312; doi:10.1186/s12889-021-10915-5)
Supplement: Supplementary file 1 — Additional file 1. Food Rx Qualitative Interview Guide. English language version of the interview guide [file 12889_2021_10915_MOESM1_ESM.pdf]

FoodRx Qualitative Interview Guide

1. Can you talk about how your experience was with the Food as Medicine Study?

Probe: What did you like about the Food as Medicine Study?
Probe: What did you dislike about the Food as Medicine Study?

3. Can you talk about any your experience with receiving the food deliveries from Dig Deep Farms?

Probe: Did the delivers arrive when you expected them?
Probe: When you were not at home, were the deliveries left at a place where you felt comfortable (i.e., your front door, leasing complex office, etc.)?

4. What was the reaction to the vegetables and/or whole grain foods that you and your family had never eaten?

Probe: How did you decide whether or not you were going to prepare a vegetable or whole grain that your family had never eaten?
Probe: How did you decide how you were going to prepare the vegetable or whole grain (i.e., preparation/cooking method)?

5. Were there any vegetables or whole grain foods that you and your family did not eat? If so, what did you do with them?

Probe: Did you give them to a family/friend?
Probe: Did you throw the food away?
Probe: Are the foods still at your home?
Probe: Do you have any suggestions or ideas about how to avoid wasting foods that go uneaten?

6. How was your experience with getting phone calls and text messages from our team?

Probe: Did you find getting phone calls from the team helpful to you?
Probe: Did you receive text messages with links to recipes and cooking ideas? Did you learn anything from these text messages?

7. Did you attend any of the cooking education classes?

(If attended classes)

What was your experience with the classes like?

Probe: What did you learn from the classes?
Probe: Who attended the classes? (You, your child, someone else?)
Probe: What did you like (or not like) about the classes?
Probe: How could we improve the cooking education classes?

(If did not attend class)

What was the reason (or reasons) that you did not attend any cooking education classes?

Probe: Was transportation an issue? Were the times of the classes an issue?
Probe: What would have helped you attend cooking classes?

8. Can you think of any suggestions about how we improve the Food as Medicine Study for the future?
